# The role of KLF4 in phagocyte activation during infectious diseases

**DOI:** 10.3389/fimmu.2025.1584873

**Published:** 2025-04-16

**Authors:** Toni Herta, Aritra Bhattacharyya, Stefan Hippenstiel, Janine Zahlten

**Affiliations:** ^1^ Department of Hepatology and Gastroenterology, Charité – Universitätsmedizin Berlin, Berlin, Germany; ^2^ Berlin Institute of Health at Charité –Universitätsmedizin Berlin, BIH Biomedical Innovation Academy, BIH Charité Clinician Scientist Program, Berlin, Germany; ^3^ Department of Respiratory Medicine and Critical Care Medicine with Sleep Medicine, Charité - Universitätsmedizin Berlin, Berlin, Germany

**Keywords:** KLF4, phagocytes, macrophages, neutrophils, dendritic cells, innate immunity, transcription factor, immune regulation

## Abstract

Phagocytes, including granulocytes (especially neutrophils), monocytes, macrophages, and dendritic cells, are essential components of the innate immune system, bridging innate and adaptive immunity. Their activation and function are tightly regulated by transcription factors that coordinate immune responses. Among these, Krüppel-like factor 4 (KLF4) has gained attention as a regulator of phagocyte differentiation, polarization, and inflammatory modulation. However, its role is highly context-dependent, exhibiting both pro- and anti-inflammatory properties based on environmental signals, cellular states, and the invading pathogen. KLF4 influences monocyte-to-macrophage differentiation and shapes macrophage polarization, promoting either inflammatory or regulatory phenotypes depending on external cues. In neutrophils, it affects reactive oxygen species production and immune activation, while in dendritic cells, it regulates monocyte-to-dendritic cell differentiation and cytokine secretion. Its diverse involvements in immune responses suggests that it contributes to maintaining a balance between effective pathogen defense and the prevention of excessive and potentially harmful inflammation. This review summarizes current knowledge on the function of KLF4 in phagocytes during infections, highlighting its regulatory mechanisms, context-dependent roles, and its impact on immune activation and resolution. Additionally, potential implications for therapeutic interventions targeting KLF4 are discussed.

## 1 Introduction

Phagocytes, such as neutrophilic granulocytes, monocytes macrophages, and dendritic cells, are critical effectors of the innate immune system and play a pivotal role in host defense against pathogens. These cells employ a diverse array of mechanisms, including phagocytosis, production of reactive oxygen species (ROS), and release of antimicrobial peptides, to eliminate invading microorganisms (1, 2). Moreover, phagocytes serve as a crucial link between innate and adaptive immunity by presenting antigens and secreting cytokines that modulate T and B cell responses (3, 4).

The activation and function of phagocytes are tightly regulated by a complex network of transcription factors that orchestrate gene expression programs in response to environmental triggers, ensuring effective pathogen elimination on the one hand, while protecting against excessive and self-destructive inflammation on the other. Among these, the transcription factor Krüppel-like factor 4 (KLF4) has received increasing attention as a regulator of several important aspects of phagocyte function, including differentiation, polarization, pathogen recognition, and the modulation of inflammatory responses. KLF4, a member of the KLF family of zinc finger transcription factors, was initially identified as a key regulator of a functional epithelial barrier (5, 6). It later gained prominence with the discovery that its expression, in combination with other transcription factors, can reprogram differentiated cells into pluripotent stem cells (7, 8). Its role in regulating the immune response has been recognized relatively recently. The function and regulation of KLF4 in phagocytes is complex, partially contradictory, and still far from fully understood. KLF4 plays a context-dependent role in infectious diseases, exhibiting both pro- and anti-inflammatory effects, depending on a multitude of factors such as context-dependent binding partners, the pathogen, and the cell type in which it is expressed (5, 9, 10).

This review aims to summarize current knowledge on the multifaceted roles of KLF4 in phagocytes during infectious diseases. The regulation and function of KLF4 in different types of phagocytes, as well as its role in response to specific pathogens, including bacterial, fungal, and parasitic agents, are examined. Furthermore, future perspectives, such as the therapeutic potential of targeting KLF4 to modulate phagocyte-mediated responses in the treatment of infectious diseases, are discussed.

## 2 Monocytes and macrophages

Monocytes are blood-circulating immune cells and precursors of tissue-resident macrophages and certain dendritic cells (11). They circulate for 1-3 days before differentiating and migrating into tissues, where they function as macrophages for weeks to months (12, 13). Macrophages exhibit functional plasticity. While traditionally the differentiated M0 naïve macrophages were classified into pro-inflammatory M1 and anti-inflammatory M2 states (14), this model has been expanded to reflect a spectrum of activation states with fluid transitions (15). M1 macrophages eliminate pathogens, produce ROS and nitrogen species, and secrete pro-inflammatory cytokines like interleukin (IL)-6 and IL-8, but may also contribute to tissue damage and autoimmune diseases (16, 17). In contrast, wound-healing and regulatory M2 macrophages suppress inflammation, with the latter secreting IL-10 to limit tissue damage and support repair (15, 18). KLF4 plays an important role at various levels of monocyte and macrophage differentiation and activation. First, KLF4 is important for monocyte-to-macrophage differentiation. It is a downstream target of PU.1, a key transcription factor for the regulation of myeloid cell fate (19), which binds to and activates the KLF4 promoter in monocytes (20). KLF4 promotes the expression of monocyte-specific markers such as cluster of differentiation (CD) 14 and CD11b and induces morphological and functional characteristics of mature monocytes (21, 22). Loss of KLF4, as demonstrated in mouse knockout models, results in a loss or marked reduction of bone marrow derived inflammatory monocytes and resident monocytes, coupled with increased apoptosis and impaired expression of key trafficking molecules (23).

Second, KLF4 is essential for macrophage polarization upon inflammatory stimuli. The rapid onset and timely resolution of inflammatory responses in macrophages are interconnected processes, both essential for effective host defense against pathogenic infections while simultaneously avoiding unnecessary collateral tissue damage. KLF4 expression is strongly upregulated in macrophages in response to inflammatory stimuli, such as pro-inflammatory cytokines, lipopolysaccharide (LPS), or pathogen contact (24–27). Several studies in murine macrophages suggest that KLF4 predominantly promotes the M2 anti-inflammatory macrophage phenotype through transcriptional synergy with signal transducer and activator of transcription (STAT) 6 and peroxisome proliferator-activated receptor gamma (PPAR-γ), driving the expression of M2 markers such as arginase 1 (ARG1) and CD206 (27–31). Moreover, KLF4 was shown to actively suppress the M1 pro-inflammatory phenotype by interfering with nuclear factor kappa-light-chain-enhancer of activated B cells (NF-κB) recruitment to pro-inflammatory gene promoters, thereby attenuating transcription of mediators like inducible nitric oxide synthase (iNOS) and tumor necrosis factor (TNF)-α (24, 32, 33). However, under specific conditions, mechanisms such as sentrin-specific protease 1 (SENP1)-mediated de-SUMOylation of KLF4 shifts its function to enhance M1 polarization via NF-κB activation, leading to increased production of pro-inflammatory cytokines (34). In line with this observation, the induction of KLF4 expression in macrophages by pro-inflammatory stimuli like self-derived interferon gamma (IFN-γ), TNF-α and bacterial-derived LPS can lead to an interaction of KLF4 with NF-κB, promoting the M1 phenotype with increased inducible nitric-oxide synthase (iNOS) expression (25).

Also, the timing within the course of an infection may influence whether KLF4 acts in a pro- or anti-inflammatory manner in macrophages. During infection, LPS-induced KLF4 expression can promote IL-10 release, particularly in the early course of infection, while later it may contribute to the release of high mobility group box 1 protein (HMGB1), a rather pro-inflammatory mediator (35). Moreover, KLF4 is upregulated alongside STAT1 in exhausted monocytes after repetitive challenges with high-dose LPS, leading to a gene expression profile indicative of both pathogenic inflammation and immunosuppression, characteristic for exhausted monocytes in septic patients (36, 37). Additionally, KLF4 interacts with non-coding RNAs to finetune macrophage responses. Micro ribonucleic acid (miR)-34a inhibits KLF4 expression, promoting an M1 phenotype, while miR-126 and exosomal miR-103-3p enhance the role of KLF4 in M2 polarization (38, 39).

It is therefore undisputed that KLF4 plays a crucial role in macrophage differentiation upon inflammatory stimuli, seemingly exhibiting a Janus-like function with both pro- and anti-inflammatory properties. Based on the available data, currently no definitive conclusion can be drawn as to whether KLF4 acts predominantly in a pro- or anti-inflammatory manner in macrophages, nor at what time point or which factors drive this behavior.

Interestingly, KLF4 plays a crucial role in maintaining rhythmic immune responses in macrophages, which are disrupted during aging. In aged macrophages, KLF4 expression is diminished, leading to a loss of circadian gene transcription and impaired functions like monocyte trafficking, phagocytosis, and bacterial resistance. This decline in KLF4-driven rhythmicity probably contributes to the age-associated vulnerability to infections and inflammatory diseases in older individuals (40).

### 2.1 KLF4 in macrophages during bacterial infections

KLF4 plays a critical role in regulating macrophage responses during bacterial infections. In *Streptococcus pneumoniae* infections, KLF4 expression in macrophages is induced only by viable pneumococci that establish direct contact with host cells and release autolysin N-acetylmuramoyl-L-alanine amidase (LytA)-dependent DNA, partially mediated via Toll-like receptor (TLR) 9 and the adaptor protein myeloid differentiation primary response 88 (MyD88) (26). This KLF4 induction promotes a pro-inflammatory macrophage phenotype, increasing cytokine secretion such as IL-6 and TNF-α while suppressing IL-10 release (26). In pneumococcal pneumonia, KLF4 in myeloid cells is essential for an effective early immune response as its deficiency leads to reduced pro-inflammatory cytokine levels, impaired bacterial clearance, and increased disease severity in the early course of infection (9). Contact with *Pseudomonas aeruginosa* also leads to an upregulation of KLF4 expression in lung resident alveolar macrophages (41). However, in contrast to *Streptococcus pneumoniae*, this upregulation does not enhance the immune response but instead promotes a pro-efferocytosis phenotype shift, a process by which apoptotic cells are removed by phagocytes, preventing severe inflammatory injury (41). Additionally, KLF4 is involved in macrophage polarization during *Mycobacterium tuberculosis* infection. Infection with *Mycobacterium tuberculosis* strongly induces KLF4 expression, leading to M2 polarization with decreased expression of antibacterial effectors such as iNOS and impaired trafficking of *Mycobacterium tuberculosis* to lysosomes, thereby promoting bacterial survival (42). Furthermore, in *Mycobacterium bovis* infection, KLF4 contributes to immune evasion by repressing major histocompatibility complex (MHC)-II expression through epigenetic modifications, thereby limiting antigen presentation and adaptive immune activation (43). Overall, KLF4 exerts both pro- and anti-inflammatory effects in bacterial infections, depending on the pathogen, probably cell type (bone marrow derived versus resident macrophages) and immune context. In *Streptococcus pneumoniae* infections, KLF4 enhances early inflammation and bacterial clearance, whereas in *Pseudomonas aeruginosa* infections, it prevents excessive immune activation, and in Mycobacterium infections, it promotes bacterial persistence.

### 2.2 KLF4 in macrophages during parasitic infections

Also in parasitic infections, KLF4 has been shown to be involved in macrophage activation. KLF4 is downregulated in the liver during *Schistosoma japonicum* infection in mice, which fosters the M2 activation of liver-resident macrophages and supports the development of liver fibrosis (44, 45). In *Echinococcus granulosus* infection, by contrast, KLF4 is upregulated in peritoneal macrophages, promoting the development of an anti-inflammatory M2 phenotype, likely as a mechanism for *Echinococcus granulosus* to evade the immune response to enhance its survival and growth (46). In cerebral malaria, infection with *Plasmodium berghei* ANKA leads to platelet-dependent upregulation of KLF4 in macrophages, resulting in increased production of pro-inflammatory cytokines such as IL-6, which probably worsens disease severity and the overall outcome (47). These findings highlight the context-dependent role of KLF4 in parasitic infections, where it can either contribute to immune evasion and tissue pathology or exacerbate inflammatory responses.

## 3 Neutrophilic granulocytes

Neutrophilic granulocytes are part of the first line of defense against bacterial infections. They rapidly migrate to infection sites, where they eliminate pathogens through phagocytosis, degranulation of granules containing a multitude of antimicrobial and cytotoxic substances, and the release of neutrophil extracellular traps (48, 49). There are generally few studies on KLF4 in neutrophilic granulocytes, limiting our understanding of its role in this cell type. As in macrophages, KLF4 expression in neutrophilic granulocytes is induced by pro-inflammatory stimuli such as LPS (50, 51). KLF4 deficiency in these cells leads to impaired degranulation of neutrophilic granules in response to LPS stimulation and delays neutrophil apoptosis, which is critical for the resolution of neutrophil-mediated inflammation (50). This impairment appears to be at least partially caused by disrupted LPS signaling through TLR4, the IκB kinase complex, and NF-κB in these cells. KLF4 seems to be essential for the proper function of this cascade in neutrophils (50). Thus, KLF4 expression in neutrophils appears to be necessary both for the effective elimination of pathogens and for limiting immune activation to prevent excessive immune responses.

### 3.1 KLF4 in neutrophilic granulocytes during bacterial infections

Our understanding of KLF4 in neutrophils during bacterial infections is primarily based on two studies. The absence of KLF4 leads to reduced intracellular killing of *Escherichia coli* in neutrophils due to impaired ROS production (50). The resulting increased susceptibility to infections, such as *Escherichia coli*, in myeloid-specific conditional KLF4-deficient mice is, however, accompanied by a reduced risk of excessive systemic inflammation and septic shock in response to *Escherichia coli* infection in these animals (50). In *Streptococcus pneumoniae* infection, KLF4 expression is upregulated in neutrophils through direct contact with viable pneumococci (52). KLF4 is necessary for effective intracellular killing of *Streptococcus pneumoniae*, enhances the production of the pro-inflammatory cytokines TNF-α and keratinocyte chemoattractant, and inhibits the production of the anti-inflammatory IL-10 (52). In summary, the upregulation of KLF4 in neutrophilic granulocytes appears to be necessary for effective clearance of bacterial pathogens.

## 4 Dendritic cells

Dendritic cells (DCs) are antigen-presenting cells that bridge the innate and adaptive immune system. They capture, process, and present antigens to T cells, thereby orchestrating immune responses (53, 54). DCs exist in various subsets, including conventional DCs, plasmacytoid DCs, and monocyte-derived DCs, each with specialized functions (55, 56). KLF4 is important for the differentiation and function of DCs, exhibiting context-dependent regulatory effects. During monocyte-to-DC differentiation, KLF4 expression is maintained in monocyte-derived DCs and inflammatory DC subsets but is repressed in Langerhans cells, a specialized DC population in the skin (55–58). Notch signaling has been identified as a key mechanism repressing KLF4 expression to allow Langerhans cell differentiation (58). Functionally, KLF4 regulates cytokine production in DCs, particularly IL-6 expression, through both direct promoter activation and epigenetic modifications (59, 60). It is also essential for interferon regulatory factor 4 (IRF4)-expressing DCs that drive T helper 2 (Th2) responses, as KLF4 deletion in these cells impairs Th2 polarization in response to parasites or allergens like house dust mites (61, 62). In fungal infections such as *Aspergillus fumigatus* and *Candida albicans*, KLF4 expression is downregulated in DCs, primarily through TLR2 activation, contrasting with its upregulation by bacterial stimuli such as LPS, which primarily activates TLR4. While both pathways result in IL-6 release, LPS-induced IL-6 production is considerably more pronounced compared to fungal stimulation (59).

## 5 Concluding remarks and future perspectives

**Figure 1:**
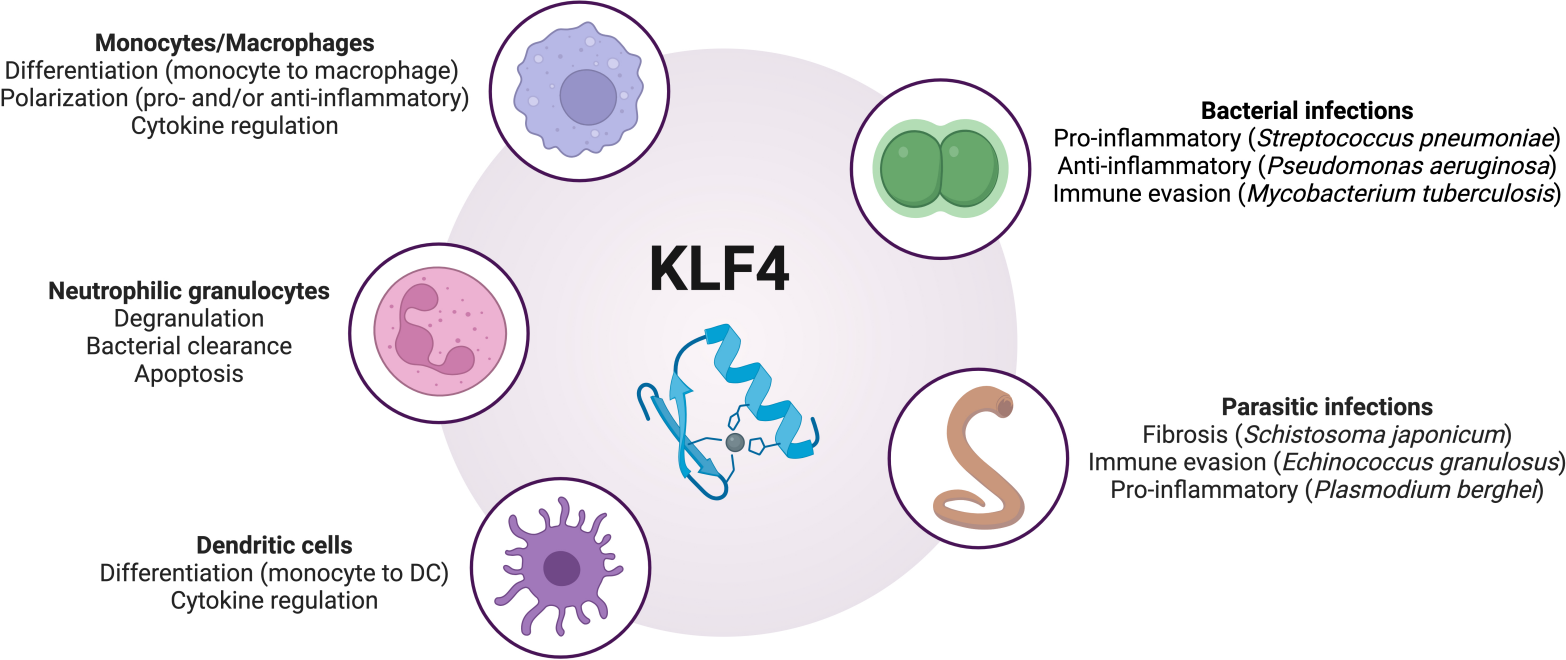
Overview of the regulatory roles of KLF4 in phagocytes during infections. DC, dendritic cells; KLF4, Krüppel-like factor 4. Image created with BioRender.

KLF4 plays a fundamental role in shaping the immune response by regulating various aspects of phagocyte function. As a key modulator of monocyte and macrophage differentiation, neutrophil activity, and dendritic cell responses, KLF4 influences how the innate immune system responds to infections (summarized in **Figure 1** and **Table 1**). Its effects extend beyond simple activation or suppression, as it seems dynamically adapt to different environmental cues, cellular states, and pathogen-specific interactions. Its role in immune responses is highly context-dependent. In some settings, it enhances inflammatory pathways to promote pathogen clearance, while in others, it promotes a shift toward anti-inflammatory states to prevent excessive tissue damage. KLF4’s ability to interact with a multitude of other transcription factors, signaling pathways, and non-coding RNAs adds another layer of complexity to its regulatory functions, suggesting that our understanding of the role and function of KLF4 in the context of inflammation and infection is still preliminary. Future studies should focus on elucidating the mechanisms and binding partners that determine how KLF4 switches between pro- and anti-inflammatory activity or between transcriptional activation and inhibition of target genes, depending on external factors. It should also be investigated whether the induction of KLF4 in other non-phagocytic cell types, through cell-cell interactions, influences its function or expression in phagocytes. In addition to investigating KLF4’s role in further bacterial, fungal, and parasitic infections, future studies should also explore its function in phagocytes during viral infections, as these cells can, under certain circumstances, serve both as viral targets, as shown for respiratory phagocytes in influenza A infection, and as mediators of antiviral immune responses (63–67). Interestingly, in this context, KLF4 is known to transactivate tripartite motif-containing protein 29 (TRIM29) expression (68), a transcription factor that modulates antiviral immune responses in various cell types (69–72). However, it remains unclear whether an interplay between KLF4 and TRIM29 also influences antiviral immune responses in phagocytes. Given the above discussed role of KLF4 in parasitic infections, a potential role of KLF4 in eosinophil granulocytes should also be explored, as these cells are fundamentally important for parasite defense (73), but the expression and function of KLF4 in these phagocytes remain unknown.

**Table 1:** Overview of the role of KLF4 in regulating the inflammatory response across different phagocyte types in response to specific pathogens, with potential therapeutic implications.

Phagocyte type	Pathogen	KLF4 function	Clinical implication	Potential therapeutic approach
Macrophages	*Streptococcus pneumoniae*	Induces pro-inflammatory cytokines, suppresses IL-10 (9, 26)	Enhances early immune response, but may promote excessive inflammation	Controlled KLF4 modulation to balance clearance vs. inflammation
	*Pseudomonas aeruginosa*	Promotes efferocytosis and anti-inflammatory response (41)	Protects against tissue damage through immune modulation	KLF4 induction to limit immunopathology
	*Mycobacterium tuberculosis*	Induces M2 polarization, reduces bactericidal activity (42)	Facilitates pathogen persistence	KLF4 inhibition to restore antimicrobial functions
	*Mycobacterium bovis*	Represses MHC-II, reduces adaptive immune activation (43)	Facilitates pathogen persistence	KLF4 inhibition to restore antimicrobial functions
	*Schistosoma japonicum*	Downregulation promotes M2 phenotype in hepatic macrophages (44, 45)	Contributes to liver fibrosis	KLF4 induction to prevent fibrotic remodeling
	*Plasmodium berghei* (cerebral malaria)	Platelet-dependent upregulation leads to IL-6 production (47)	Exacerbates inflammation and worsens disease outcome	KLF4 inhibition to reduce inflammatory severity
Neutrophilic granulocytes	*Escherichia coli*	Enhances ROS production (50)	Improves pathogen clearance, but increases risk of septic shock	Controlled KLF4 modulation to balance clearance vs. inflammation
	*S. pneumoniae*	Enhances bacterial killing and pro-inflammatory cytokine production, suppresses IL-10 (52)	Improves pathogen clearance	Controlled KLF4 modulation to balance clearance vs. inflammation
Dendritic Cells	*Candida albicans, Aspergillus fumigatus*	Downregulated by TLR2 signaling; reduces IL-6 production (59)	Weakens antifungal immunity	KLF4 induction to enhance antigen presentation
	*Parasites*	Promotes Th2 polarization via IRF4+ dendritic cells (61)	Enhances anti-parasitic responses	KLF4 induction to promote adaptive immune response

IL, interleukin; IRF4, interferon regulatory factor 4; KLF4, Krüppel-like factor 4; MHC, major histocompatibility complex; ROS, reactive oxygen species; TLR, Toll-like receptor.

Given its pivotal regulatory role in phagocyte function, KLF4 presents as an interesting, yet complex, candidate for therapeutic intervention in infectious diseases. Modulating KLF4 activity could allow for tailored immune responses - enhancing its expression may promote anti-inflammatory macrophage polarization or efferocytosis to prevent tissue damage in chronic or excessive inflammation, while inhibiting KLF4 could restore bactericidal functions in macrophages or enhance pro-inflammatory responses during immune suppression. Similarly, selective KLF4 modulation in neutrophils and dendritic cells could influence microbial clearance and adaptive immune activation, respectively. However, due to its context-dependent and time-sensitive functions, any therapeutic approach must be precisely timed and cell-specific to avoid adverse effects, such as impaired pathogen clearance or immunopathology. Moreover, concerns regarding KLF4’s role in tumorigenesis (74–77) underscore the need for cautious and highly targeted strategies in potential future therapeutic development. We identified one study that used honokiol, a natural compound derived from parts of the plant *Magnolia grandiflora* commonly used in Oriental herbal medicine, to counteract LPS-induced upregulation of KLF4 in murine microglial cells, resulting in reduced production of proinflammatory cytokines (78). However, whether this approach allows for a targeted and finely tunable modulation of KLF4 *in vivo* remains uncertain.

While this review summarizes the current understanding of KLF4 in phagocyte activation during infectious diseases, several limitations should be acknowledged. First, the existing body of literature is limited, with most experimental data derived from murine models or *in vitro* systems, making translational applicability to human disease uncertain. Second, although one study has explored a potential therapeutic strategy targeting KLF4, further investigations in the context of infectious diseases are still lacking. Thus, the therapeutic strategies discussed remain largely theoretical. Third, due to the fragmented and pathogen-specific nature of the available data, comprehensive comparisons across different infectious agents or phagocyte subtypes are difficult, and conclusions may not be generalizable.

Therefore, while KLF4 represents a significant regulator of immune function in phagocytes, its complex and context-dependent roles necessitate a cautious approach when considering it as a therapeutic target, underscoring the need for further in-depth studies to fully understand its implications in immunity and disease.
